# *Paenibacillus gyeongsangnamensis* sp. nov., Isolated from Soil

**DOI:** 10.4014/jmb.2404.04038

**Published:** 2024-06-17

**Authors:** Hyosun Lee, Dhiraj Kumar Chaudhary, Dong-Uk Kim

**Affiliations:** 1 Department of Biological Science, College of Science and Engineering, Sangji University, Wonju 26339, Republic of Korea; 2 Department of Microbiology, Pukyong National University, Busan 48513, Republic of Korea

**Keywords:** *Paenibacillus gyeongsangnamensis* sp. nov., soil, *Paenibacillaceae*, taxonomy, phylogeny

## Abstract

A Gram-stain-positive, aerobic, white-coloured, rod-shaped bacteria, designated as a strain dW9^T^, was isolated from soil. Strain dW9^T^ was catalase-positive and oxidase-negative. Strain dW9^T^ grew at temperature of 20–37°C and at pH of 5.0–7.0. Phylogenetic and 16S rRNA gene analysis indicated that strain dW9^T^ belonged to the genus *Paenibacillus* with its closest relative being *Paenibacillus filicis* S4^T^ (97.4% sequence similarity). The genome size of dW9^T^ was 7,787,916 bp with DNA G+C content of 51.3%. The digital DNA–DNA hybridization (dDDH) and average nucleotide identity (ANI) values of dW9^T^ with its closest relatives were found to be <22.0% and <74.0%, respectively. The only respiratory quinone was MK-7, and the major fatty acids were antiso-C_15:0_ and iso-C_16:0_. Overall, the comprehensive taxonomic analysis revealed that strain dW9^T^ m*et al*l the fundamental criteria to be classified as a novel species within the genus *Paenibacillus*. Accordingly, we propose the name *Paenibacillus gyeongsangnamensis* sp. nov., with the type strain dW9^T^ (=KCTC 43431^T^ =NBRC 116022^T^).

## Introduction

The genus *Paenibacillus* was initially established by Ash *et al*. in 1993 for the taxonomic classification of 16S rRNA group 3 bacilli [1]. Subsequently, various species originally classified under the genus *Bacillus* were reassigned to the genus *Paenibacillus* [2, 3]. The type species of this genus is *P. polymyxa*. At present, *Paenibacillus* is categorized under the family *Paenibacillaceae*, which belongs to the phylum *Bacillota*. This genus currently includes 399 species, 304 of which are validly published with correct names (accession date: April 04, 2024; https://lpsn.dsmz.de/genus/paenibacillus). *Paenibacillus* species have been obtained from various sources, including soil, air, sediment, eutrophic lake, hot spring, freshwater, mountain, rhizosphere, phyllosphere, plant, seed, food, gut, insect, necrotic wound, and fecal samples [3-14]. This study characterized and determined the taxonomic status of strain dW9^T^ in the genus *Paenibacillus*, which was isolated from a soil sample collected from the Republic of Korea.

## Materials and Methods

### Isolation of Strains

Strain dW9^T^ was isolated from a soil sample collected from Gyeongsangnam in the Republic of Korea (35°28'48.0''N 128°13'12.0''E). The strain was isolated by the standard dilution plating technique using R2A media (MB Cell, Republic of Korea). After plating, the Petri dishes were placed in an incubator at 25°C for 7 days. Subsequently, white colonies were selected and repeatedly streaked on R2A agar. Pure colonies of strain dW9^T^ were obtained and temporarily stored at 4°C. After the completion of taxonomic analyses, strain dW9^T^ was preserved in glycerol stocks at −80°C and was submitted to the Korean Collection for Type Cultures and NITE Biological Resource Center.

### 16S rRNA Gene Sequence and Phylogenetic Analysis

Genomic DNA from strain dW9^T^ was extracted using the HiGene Genomic DNA Prep Kit (BioFact, South Korea). PCR amplification of the 16S rRNA gene was performed using forward (27F) and reverse (1492R) primers [15]. The amplified PCR products were sequenced and analyzed as described previously [16]. The closest phylogenetically related taxa were sorted by analyzing and comparing the 16S rRNA nucleotide sequences using the EzBioCloud server [17]. Phylogenetic trees were constructed with MEGA X software [18] using the maximum likelihood (ML) [19], neighbor-joining (NJ) [20], and maximum parsimony (MP) algorithms [21]. The topologies of phylogenetic trees were estimated using the bootstrap resampling method with 1,000 replications [22]. The evolutionary distances were determined using Kimura’s two-parameter model [23].

### Genomic Analysis

The genome was sequenced by the Illumina MiSeq sequencing technique, and raw sequences were assembled using Platanus-allee v. 2.2.2 [24] and SPAdes v. 3.13.0 [25] assembly tools. The quality of the genome sequence was assessed using the ContEst16S algorithm [26] and BLAST-N tool [27]. The annotation of the assembled genome sequence was performed using the Rapid Annotations using Subsystems Technology (RAST) server [28] and the Prokaryotic Genome Annotation Pipeline (PGAP) [29]. The DNA G + C content was directly determined from the genome sequence data. Biosynthetic gene clusters (BCGs) for various secondary metabolites were explored using antiSMASH 5.0 [30]. The genomic similarities between strain dW9^T^ and reference species were calculated using the Genome-to-Genome Distance Calculator [31] and the average nucleotide identity (ANI) tool [32]. The phylogenomic tree was generated on the Type (Strain) Genome Server [33] using FastME 2.1.6.1 [34].

### Morphological, Physiological, and Biochemical Analyses

Cellular morphologies of strain dW9^T^ were analyzed by transmission electron microscopy (Talos L120C; FEI) after culturing the strain on R2A agar at 25°C for 5 days. The Gram stain reaction was determined using the Color Gram 2 Kit (bioMérieux, France). Anaerobic growth, motility, catalase, and oxidase tests were performed as described previously [16]. Endospores were examined by phase-contrast microscopy using a BX53-DIC microscope (Olympus) [35]. The temperature, pH, and NaCl ranges for growth were determined as described previously [36]. Moreover, the ability to hydrolyze cellulose, casein, DNA, starch, and Tween 80 was assessed as illustrated previously [37]. Various other biochemical, enzymatic, and carbon assimilation features were assessed using API ZYM, API 20NE, and API ID 32 GN kits (bioMérieux).

### Chemotaxonomic Characterization

Cellular fatty acid compositions were assessed after growing strain dW9^T^ and its closest reference taxa on R2A agar at 25°C for 3 days. After the late log phase of growth, the biomass of all strains was harvested and used for extracting fatty acids. The extracted fatty acids were analyzed and identified using the MIDI protocol [38]. Peptidoglycans were analyzed as described previously [39]. Quinones and polar lipids were analyzed using freeze-dried cells in accordance with previously described methods [40, 41]. Polar lipid spots on TLC plates were visualized by spraying with various reagents [42].

## Results and Discussion

**Fig. 1:**
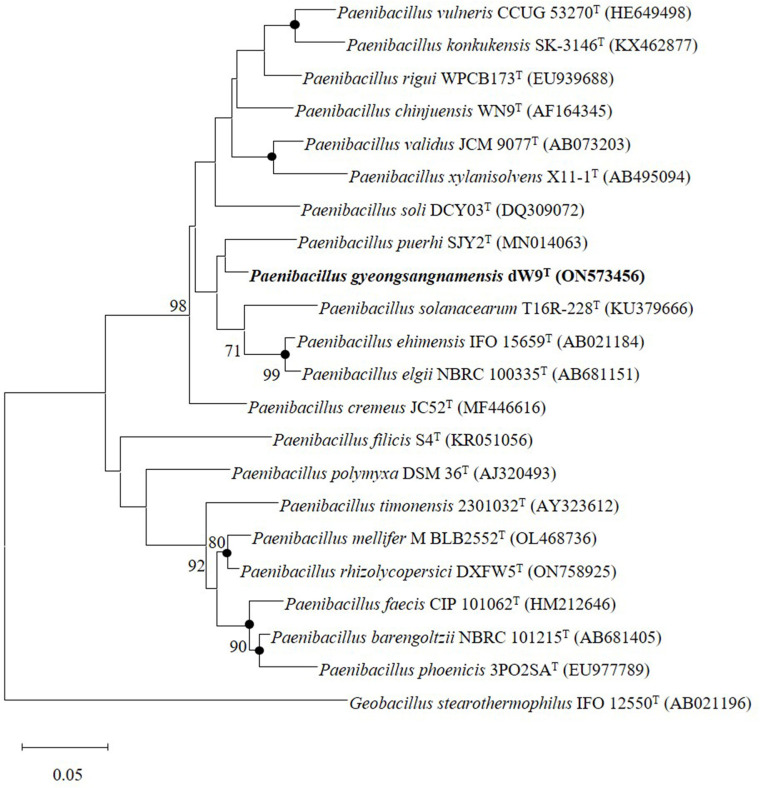
Maximum likelihood tree based on 16S rRNA gene sequences of strain dW9^T^ closest reference species. Nodes recovered by maximum-likelihood, neighbor-joining, and maximum-parsimony trees are denoted by filled circles. The numbers at branch nodes are percentage of 1,000 bootstrap replicates (values >70% are only illustrated). NCBI GenBank accession numbers for 16S rRNA gene sequences are provided in parentheses. *Geobacillus stearothermophilus* IFO 12550^T^ was used as an out-group. The scale bar indicated 0.05 substitutions per nucleotide position.

The length of the 16S rRNA gene nucleotide sequence of strain dW9^T^ was 1,447 bp. Moreover, 16S rRNA gene analysis revealed that strain dW9^T^ belonged to the genus *Paenibacillus*. Its closest phylogenetic neighbors were *P. filicis* S4^T^ (97.4%), *P. chinjuensis* WN9^T^ (97.3%), *P. validus* JCM 9077^T^ (97.1%), *P. mucilaginosus* VKPM B-7519^T^ (97.0%), *P. puerhi* SJY2^T^ (96.8%), and *P. cremeus* JC52^T^ (963.8%). The 16S rRNA gene sequence identities between strain dW9^T^ and all other phylogenetically related taxa were below the cut-off value of <98.7% for species demarcation [43, 44]. This suggested that strain dW9^T^ could be considered a novel species in the genus *Paenibacillus*. Furthermore, ML and NJ phylogenetic trees depicted that strain dW9^T^ formed a clade with *P. puerhi* SJY2^T^ (Figs. 1 and S1), whereas the MP tree revealed the formation of a clade with *P. cremeus* JC52^T^ (Fig. S2).

Quality assessment confirmed that the genome sequence generated from strain dW9^T^ was valid and contamination-free. The genome size of strain dW9^T^ was 7,787,916 bp with a DNA G + C content of 51.3%. The genome sequence of strain dW9^T^ was assembled in 71 contigs with an N50 value of 243,884 bp and genome coverage of 136.0× (Table S1). The annotated data obtained using RAST revealed 326 subsystem features in the genome of strain dW9^T^ (Fig. S3). The strain also contained numerous BGCs encoding various secondary metabolites, such as linear azol(in)e-containing peptides, type III polyketide synthase, cyclic lactone autoinducer peptide, thiopeptide, phosphonate, proteusin, and terpene (Table S2). The dDDH and ANI values between strain dW9^T^ and its closest phylogenetically related taxa ranged from 19.2% to 21.6% and 69.6% to 73.9%, respectively (Table S3). The genome relatedness values between strain dW9^T^ and its reference species were below the threshold values [dDDH (70.0%) and ANI (95.0%)], suggesting that strain dW9^T^ was genomically different from its closest members [45, 46]. Furthermore, the phylogenomic tree revealed that strain dW9^T^ formed a clade with *P. cremeus* JC52^T^ (Fig. S4).

The cells of strain dW9^T^ were rod shaped and flagellated (Fig. S5). Moreover, strain dW9^T^ was motile. Catalase and nitrate reduction tests were positive, whereas the oxidase test was negative. Strain dW9^T^ could grow at a temperature of 20–37°C and a pH of 5.0–7.0 and could tolerate 2.0% (w/v) NaCl. It could hydrolyze esculin and Tween 80. β-galactosidase and β-glucosidase activities were positive. The strain could assimilate D-glucose, L-arabinose, D-mannitol, gluconate, and D-melibiose. Other distinguishing features of strain dW9^T^ are presented in the species protologue and provided along with those of its reference species in Table 1. All enzyme activity and assimilation data obtained using API kits are provided in Table S4.

The sole respiratory quinone in strain dW9^T^ was menaquinone (MK)-7. Diphosphatidylglycerol, phosphatidylglycerol, phosphatidylmethylethanolamine, and phosphatidylethanolamine were the predominant polar lipids (Fig. S6). One unidentified polar lipid (L) was also observed. Both respiratory quinone and polar lipid profiles of strain dW9^T^ closely resembled those of its related reference taxa [12, 47]. The peptidoglycan was identified as meso-diaminopimelic acid (DAP). The key fatty acids in strain dW9^T^ were antiso-C_15:0_ (75.6%) and iso-C_16:0_ (6.8%). The key fatty acid profiles aligned with those of the closest reference taxa. However, the composition of minor fatty acids differed proportionally between strain dW9^T^ and the reference species (Table 2).

### Taxonomic Conclusion

On the basis of data presented here, we proposed strain dW9^T^ as a novel species in the genus *Paenibacillus* with the name *Paenibacillus gyeongsangnamensis* sp. nov.

### Description of *Paenibacillus gyeongsangnamensis* sp. nov.

*Paenibacillus gyeongsangnamensis* sp. nov. (gyeong.sang.na.men'sis. N.L. masc. adj. *gyeongsangnamensis*, referring to Gyeongsangnam, the place of Republic of Korea).

Cells are aerobic, Gram-stain-positive, motile, endospore-forming, rod shaped (4.9–5.1 × 1.3–1.5 μm), and flagellated. Colonies on R2A agar are white, circular (4.6–5.4 mm in diameter), and convex. Cells grow at temperature 20–37°C (optimum, 25°C), at pH 5.0–7.0 (optimum, 7.0), and at 0–2.0% NaCl concentration (optimum without NaCl). Positive for catalase and nitrate reduction tests, and negative for oxidase activity. Hydrolyse Tween 80 and esculin, but not starch, DNA, gelatin, casein, and urea. Positive for alkaline phosphatase, esterase (C4), esterase lipase (C8), leucine arylamidase, acid phosphatase, naphtol-AS-BI-phosphohydrolase, β-galactosidase, α-glucosidase, β-glucosidase, α-mannosidase, and α-fucosidase. Assimilates D-glucose, L-arabinose, D-mannitol, gluconate, salicin, D-melibiose, L-histidine, 2-ketogluconate, D-ribose, inositol, D-sucrose, and glycogen. The key fatty acids are antiso-C_15:0_ and iso-C_16:0_. The sole menaquinone is MK-7, diagnostic peptidoglycan is meso-DAP, and major polar lipids are diphosphatidylglycerol, phosphatidylglycerol, phosphatidylmethylethanolamine, and phosphatidylethanolamine. The DNA G+C content of the type strain is 51.3%.

The type strain, dW9^T^ (=KCTC 43431^T^ =NBRC 116022^T^), was isolated from soil in Republic of Korea (GPS coordinates: 35°28'48.0"N 128°13'12.0"E).

The GenBank/EMBL/DDBJ accession numbers for the 16S rRNA sequence and genome sequence of strain dW9^T^ are ON573456 and JAQAGZ000000000, respectively.

## Supplemental Materials

Supplementary data for this paper are available on-line only at http://jmb.or.kr.



## Figures and Tables

**Table 1:** Differentiating properties of dW9^T^ and closely affiliated reference taxa.

Characteristic	1	2	3	4	5	6	7
Growth temperature (°C)	20–37	15–37	18–45	15–42	15–45	15–37	15–40
Highest salt tolerance (%, w/v)	2.0	3.0	2.0	1.0	2.0	4.0	0.0
pH range	5.0–7.0	5.5–9.0	6.5–8.0	5.0–8.5	6.0–8.5	5.0–8.0	6.0–7.0
Catalase/oxidase	+/-	+/+	+/+	+/-	+/-	-/+	+/-
Motility	+	+	+	+	-	+	+
Nitrate reduction	+	-	-	+	−	+	-
Hydrolysis of							
Starch	-	+	+	+	-	+	+
Casein	-	-	+	-	-	-	+
Tween 80	+	+	+	-	-	-	+
Esculin	+	+	-	+	-	-	+
Enzyme activity							
Alkaline phosphatase	+	+	-	-	-	-	-
Leucine arylamidase	+	+	+	+	-	+	+
Valine arylamidase	-	-	-	-	-	+	+
Cystine arylamidase	-	-	-	-	-	+	+
Acid phosphatase	+	+	-	-	-	+	-
*β*-Galactosidase	+	+	-	-	+	-	-
*α*-Glucosidase	+	+	-	-	-	-	-
*β*-Glucosidase	+	+	-	+	-	-	+
*N*–Acetyl-*β*-glucosaminidase	-	+	-	-	-	-	-
*α*-Mannosidase	+	-	-	-	-	-	-
α-Fucosidase	+	-	-	-	-	-	-
Assimilation from (API 20NE and ID 32 GN test)							
D-Glucose	+	+	-	+	-	+	+
L-Arabinose	+	-	-	-	-	+	+
D-Mannose	-	+	+	w	-	-	+
D-Mannitol	+	+	-	+	-	-	+
*N*-Acetyl-D-glucosamine	-	+	-	-	-	-	-
D-Maltose	-	+	+	+	-	+	+
Gluconate	+	+	-	+	-	-	-
Salicin	+	+	-	-	-	-	+
D-Melibiose	+	+	-	+	-	-	+
L-Fucose	-	-	-	-	-	-	+
Propionate	-	-	-	+	-	-	-
L-Histidine	+	-	-	-	-	-	-
2-Ketogluconate	+	-	-	-	-	-	-
4-Hydroxy-benzoate	-	-	-	+	-	-	-
D-Ribose	+	-	-	+	-	-	-
Inositol	+	+	-	+	-	-	+
D-Sucrose	+	+	-	+	-	-	+
Acetate	-	+	-	+	-	-	-
Glycogen	w	+	-	+	-	-	-
DNA G + C content	51.3%	53.5%	(53.0 mol%)	52.2%	(53.7 mol%)	53.1%	50.75

Strains: 1, dW9^T^; 2, *P. filicis* KACC 14197^T^; 3, *P. chinjuensis* KACC 12279^T^ [48]; 4, *P. validus* KACC 14477^T^ ; 5, *P. mucilaginosus* KACC 13999^T^ [49]; 6, *P. puerhi* KCTC 43242^T^ ; 7, *P. cremeus* KACC 21221^T^. All data were generated in this study except the data provided in parentheses which were obtained from literatures. +, positive; w, weakly positive; -, negative.

**Table 2:** Cellular fatty acid profiles (% of totals) of dW9^T^ and phylogenetically related reference species.

Fatty acids	1	2	3	4	5	6	7
Saturated							
C_10:0_	–	1.0	1.6	1.3	1.0	–	1.0
C_12:0_	tr	1.0	1.6	1.3	1.1	1.0	tr
C_14:0_	1.1	1.4	2.1	1.6	1.6	1.4	2.2
C_16:0_	2.8	5.5	11.1	7.5	6.2	10.8	5.6
Unsaturated							
C_16:1_ *ω*7*c* alcohol	1.1	–	–	1.7	tr	1.5	–
C_16:1_ *ω*11*c*	1.5	–	–	2.7	5.2	1.0	–
Branched saturated							
iso-C_14:0_	4.5	6.3	1.6	3.7	2.2	3.7	3.9
iso-C_15:0_	2.6	2.6	2.3	4.6	2.7	2.5	4.1
iso-C_16:0_	**6.8**	14.1	3.6	10.3	3.9	9.8	12.1
iso-C_17:0_	–	1.0	–	1.7	–	1.1	1.6
anteiso-C_13:0_	tr	–	–	–	–	–	–
anteiso-C_15:0_	**75.6**	61.8	62.9	56.8	60.6	58.4	60.8
anteiso-C_17:0_	3.2	4.4	3.9	6.0	3.5	3.5	5.1
Hydroxy							
C_12:0_ 3OH	–	–	2.8	–	1.1	1.3	–
C_13:0_ 2OH	–	–	5.8	–	2.8	1.6	–

Strains: 1, dW9^T^; 2, *P. filicis* KACC 14197^T^; 3, *P. chinjuensis* KACC 12279^T^; 4, *P. validus* KACC 14477^T^; 5, *P. mucilaginosus* KACC 13999^T^; 6, *P. puerhi* KCTC 43242^T^ ; 7, *P. cremeus* KACC 21221^T^ . TR, trace amount (<1.0%); –, not detected.
