# Supplementary material for: Paenibacillus gyeongsangnamensis sp. nov., Isolated from Soil
Source: J Microbiol Biotechnol. 2024 Jun 17;34(8):1636–41. doi: 10.4014/jmb.2404.04038 (PMC11380503; doi:10.4014/jmb.2404.04038)
Supplement: Supplementary file 1 [file jmb-34-8-1636-supple.pdf]

J. Microbiol. Biotechnol. 2024. 34(8): 1636–1641

https://doi.org/10.4014/jmb.2404.04038

Supplementary Tables and Figures

Paenibacillus gyeongsangnamensis sp. nov., isolated from soil

Hyosun Lee [1], Dhiraj Kumar Chaudhary [2], and Dong-Uk Kim [1]

[1] Department of Biological Science, College of Science and Engineering, Sangji University, Wonju 26339, Republic of Korea

[2] Department of Microbiology, Pukyong National University, Busan 48513, Republic of Korea

*Corresponding author: Dong-Uk Kim (dukim@sangji.ac.kr)

Table S1. The genome features of strain dW9^T.

**Genome features**	**dW9^T**
Genome size (bp)	7,787,916
G + C content (%)	51.3
No. of contigs	71
N50 (bp)	243,884
L50	12
No. of subsystem	326
No. of coding sequences	8,289
Number of RNAs	116
Genome coverage	136.0x

Table S2. The distribution of biosynthetic gene clusters (BGCs) in the genome of the strain dW9^T.

dW9^T					
**Genomic regions**	**Type**	**From**	**To**	**Most similar known cluster**	**Similarity (%)**
Region 3.1	LAP	74,233	103,999		
Region 3.2	T3PKS	117,456	158,628	Sch-47554/Sch-47555	3
Region 3.3	Cyclic-lactone-autoinducer	258,582	279,145		
Region 11.1	Thiopeptide	160,213	186,708		
Region 18.1	Terpene	89,025	111,079		
Region 19.1	Phosphonate	101,417	120,914		
Region 24.1	RRE-containing	32,387	52,656		

LAP: Linear azol(in)e-containing peptides; T3PKS: Type III PKS; PKS: Polyketide synthase; RRE: Rev response element.

Table S3. Average nucleotide identity (ANI) and digital DNA-DNA hybridization (dDDH) values between strain dW9^T and phylogenetically closest members.

Reference strains	dW9^T	
	ANI (%)	dDDH (%)
dW9^T	100	100
NC1	69.7	21.6
Paenibacillus filicis KACC 14197^T	72.6	19.2
Paenibacillus validus JCM 9077^T	73.9	19.7
Paenibacillus puerhi SJY2^T	72.7	19.5
Paenibacillus cremeus JC52^T	73.9	20.4
Paenibacillus mellifer MBLB2552^T	69.6	21.0

Table S4. The enzymatic and carbon assimilation data obtained from API ZYM, API 20NE and API ID 32 GN tests of strain dW9^T and related reference members. Strains: 1, dW9^T; 2, *P. filicis* KACC 14197^T; 3, *P. chinjuensis* KACC 12279^T; 4, *P. validus* KACC 14477^T; 5, *P. mucilaginosus* KACC 13999^T; 6, *P. puerhi* KCTC 43242^T ; 7, *P. cremeus* KACC 21221^T. +, positive; w, weakly positive; -, negative.

**API ZYM test**	**1**	**2**	**3**	**4**	**5**	**6**	**7**
Alkaline phosphatase	+	+	-	-	-	-	-
Esterase (C4)	+	+	+	+	+	+	+
Esterase Lipase (C8)	+	+	+	+	+	+	+
Lipase (C14)	-	-	-	-	-	-	-
Leucine arylamidase	+	+	+	+	-	+	+
Valine arylamidase	-	-	-	-	-	+	+
Cystine arylamidase	-	-	-	-	-	+	+
Trypsin	-	-	-	-	-	+	-
α-Chymotrypsin	-	-	-	-	-	-	-
Acid phosphatase	+	+	-	-	-	+	-
Naphtol-AS-BI-phosphohydrolase	+	+	+	+	+	+	+
α-Galactosidase	-	-	-	-	-	-	-
β-Galactosidase	+	+	-	-	+	-	-
β-Glucuronidase	-	-	-	-	-	-	-
α-Glucosidase	+	+	-	-	-	-	-
β-Glucosidase	+	+	-	+	-	-	+
N–Acetyl -β-glucosaminidase	-	+	-	-	-	-	-
α-Mannosidase	+	-	-	-	-	-	-
α-Fucosidase	+	-	-	-	-	-	-
**API 20NE test**							
Reduction of NO_3^- to NO_2^-	+	-	-	+	-	+	-
Reduction of NO_3^- to N_2	-	-	-	-	-	-	-
Indole production	-	-	-	-	-	-	-
Glucose Acidification	-	-	-	-	-	-	-
Arginine dihydrolase	-	-	-	-	-	-	-
Urease	-	-	-	-	-	-	-
β-Glucosidase (esculin hydrolysis)	+	+	-	+	-	-	+
Protease (gelatin hydrolysis)	-	-	+	-	-	-	-
β-Galactosidase (PNPG)	+	+	-	-	+	+	+
D-Glucose	+	+	-	+	-	+	+
L-Arabinose	+	-	-	-	-	+	+
D-Mannose	-	+	+	w	-	-	+
D-Mannitol	+	+	-	+	-	-	+
N-Acetyl-D-glucosamine	-	+	-	-	-	-	-
D-Maltose	-	+	+	+	-	+	+
Gluconate	+	+	-	+	-	-	-
Caprate	-	-	-	-	-	-	-
Adipate	-	-	-	-	-	-	-
Malate	-	-	-	-	-	-	-
Citrate	-	-	-	-	-	-	-
Phenyl-acetate	-	-	-	-	-	-	-
**API ID 32 GN test**							
D-Mannitol	+	+	-	+	-	-	+
D-Glucose	+	+	-	+	-	+	+
Salicin	+	+	-	-	-	-	+
D-Melibiose	+	+	-	+	-	-	+

L-Fucose	-	-	-	-	-	-	+
D-Sorbitol	-	-	-	-	-	-	-
L-Arabinose	+	-	-	-	-	+	+
Propionate	-	-	-	+	-	-	-
Caprate	-	-	-	-	-	-	-
Valerate	-	-	-	-	-	-	-
Citrate	-	-	-	-	-	-	-
L-Histidine	+	-	-	-	-	-	-
2-Ketogluconate	+	-	-	-	-	-	-
3-Hydroxy-butyrate	-	-	-	-	-	-	-
4-Hydroxy-benzoate	-	-	-	+	-	-	-
L-Proline	-	-	-	-	-	-	-
L-Rhamnose	-	-	-	-	-	-	-
N-Acetyl-D-glucosamine	-	+	-	-	-	-	-
D-Ribose	+	-	-	+	-	-	-
Inositol	+	+	-	+	-	-	+
D-Sucrose	+	+	-	+	-	-	+
D-Maltose	-	+	+	+	-	+	+
Itaconate	-	-	-	-	-	-	-
Suberate	-	-	-	-	-	-	-
Malonate	-	-	-	-	-	-	-
Acetate	-	+	-	+	-	-	-
Lactate	-	-	-	-	-	-	-
L-Alanine	-	-	-	-	-	-	-
5-Ketogluconate	-	-	-	-	-	-	-
Glycogen	w	+	-	+	-	-	-
3-Hydroxy-benzoate	-	-	-	-	-	-	-
L-Serine	-	-	-	-	-	-	-

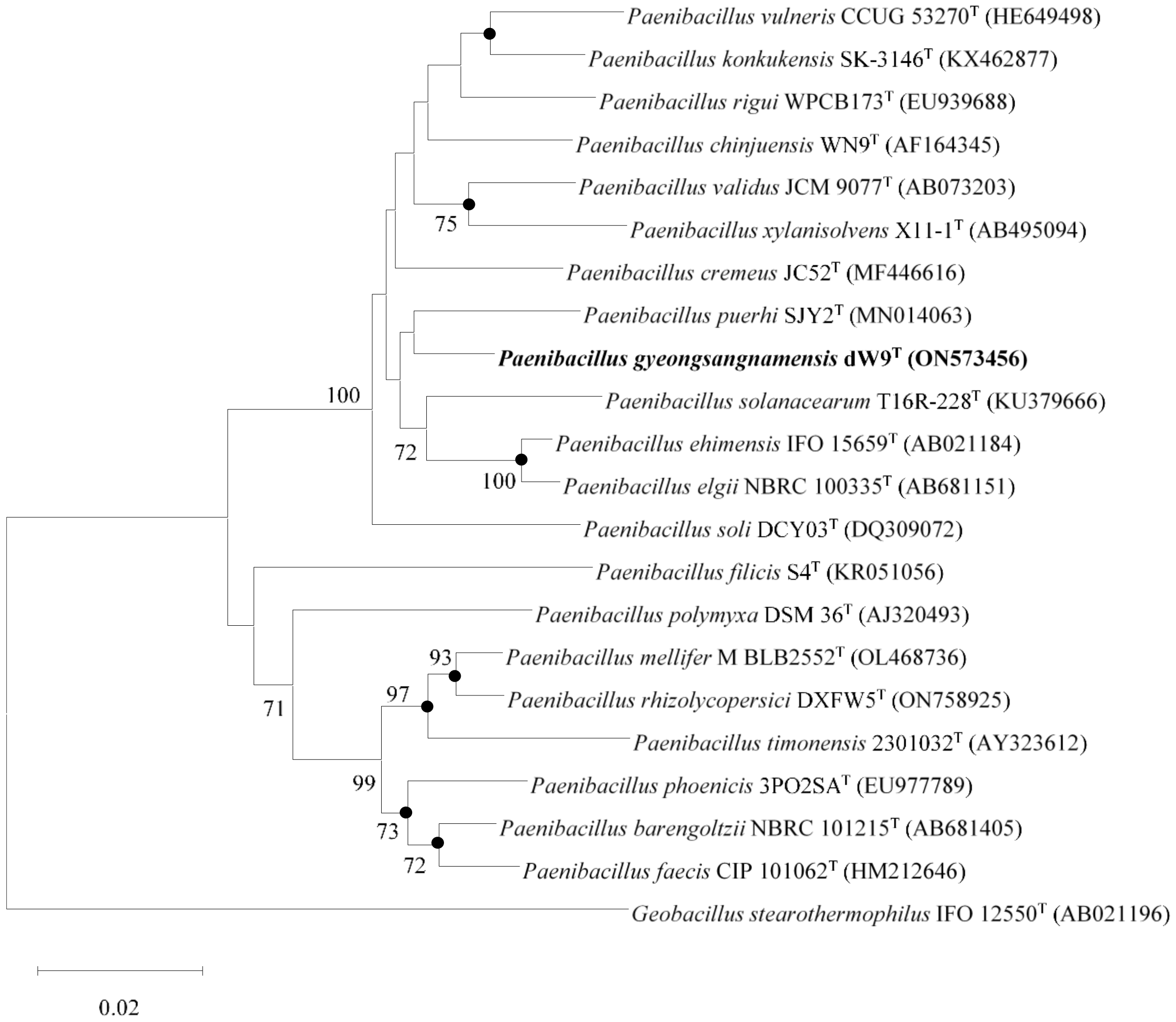

Fig. S1. Neighbour-joining tree based on 16S rRNA gene sequences of strain dW9^{T} and closest reference species. Nodes recovered by maximum-likelihood, neighbor-joining, and maximum-parsimony trees are denoted by filled circles. The numbers at branch nodes are percentage of 1,000 bootstrap replicates (values >70% are only illustrated). NCBI GenBank accession numbers for 16S rRNA gene sequences are provided in parentheses. *Geobacillus stearothermophilus* IFO 12550^{T} was used as an out-group. The scale bar indicated 0.02 substitutions per nucleotide position.

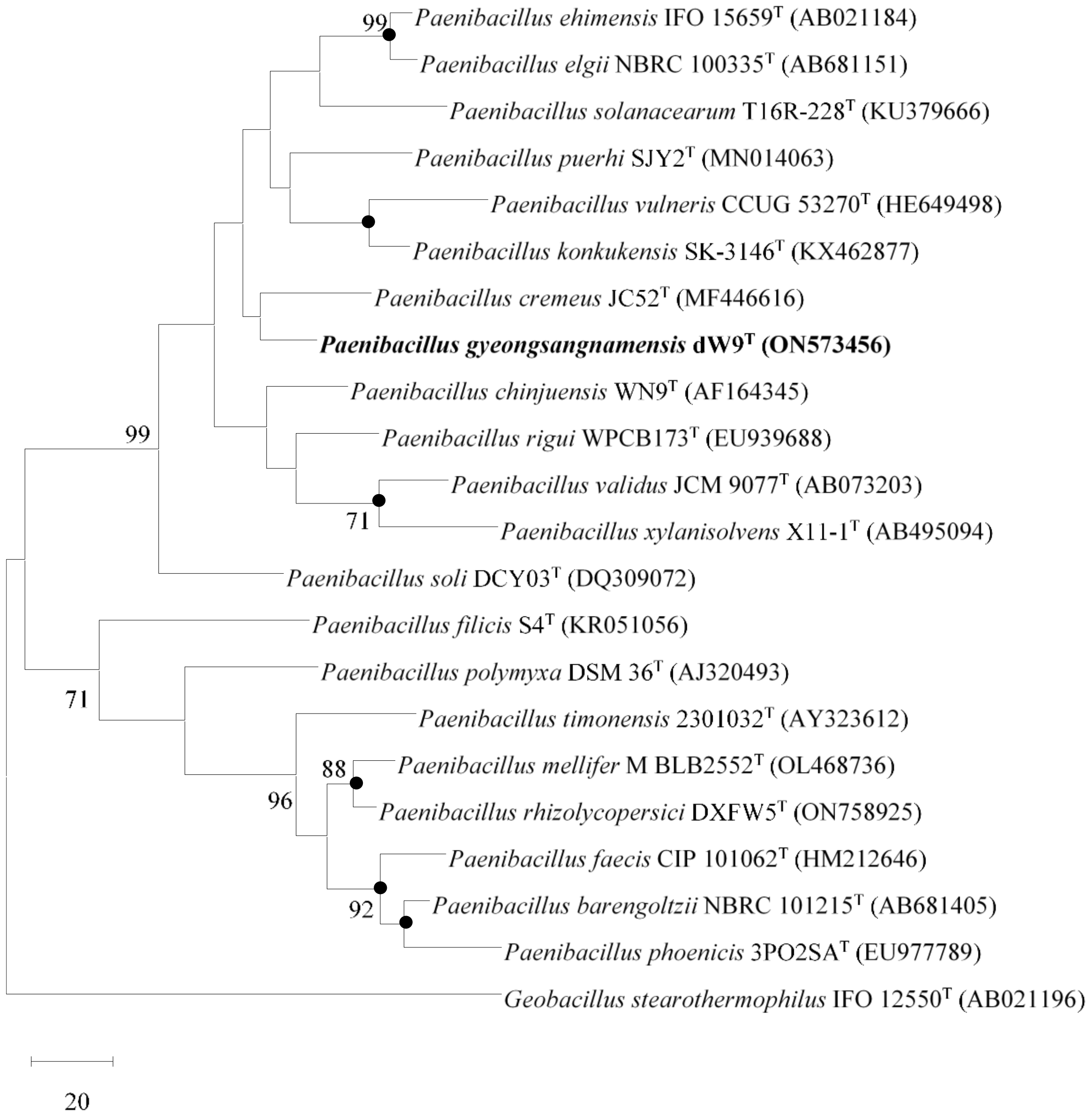

Fig. S2. Maximum-parsimony tree based on 16S rRNA gene sequences of strain dW9^T and closest reference species. Nodes recovered by maximum-likelihood, neighbor-joining, and maximum-parsimony trees are denoted by filled circles. The numbers at branch nodes are percentage of 1,000 bootstrap replicates (values >70% are only illustrated). NCBI GenBank accession numbers for 16S rRNA gene sequences are provided in parentheses. *Geobacillus stearothermophilus* IFO 12550^T was used as an out-group. The scale bar indicated 20 substitutions per nucleotide position.

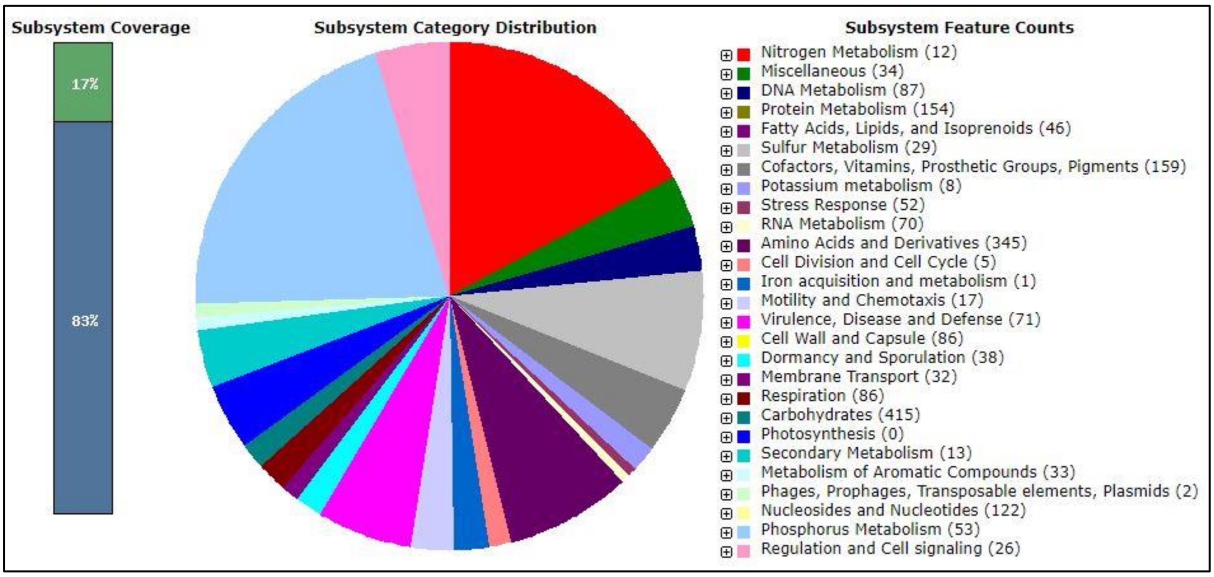

Fig. S3. Genome annotation of strain dW9^{T} performed by RAST (Rapid Annotation using Subsystem Technology) server.

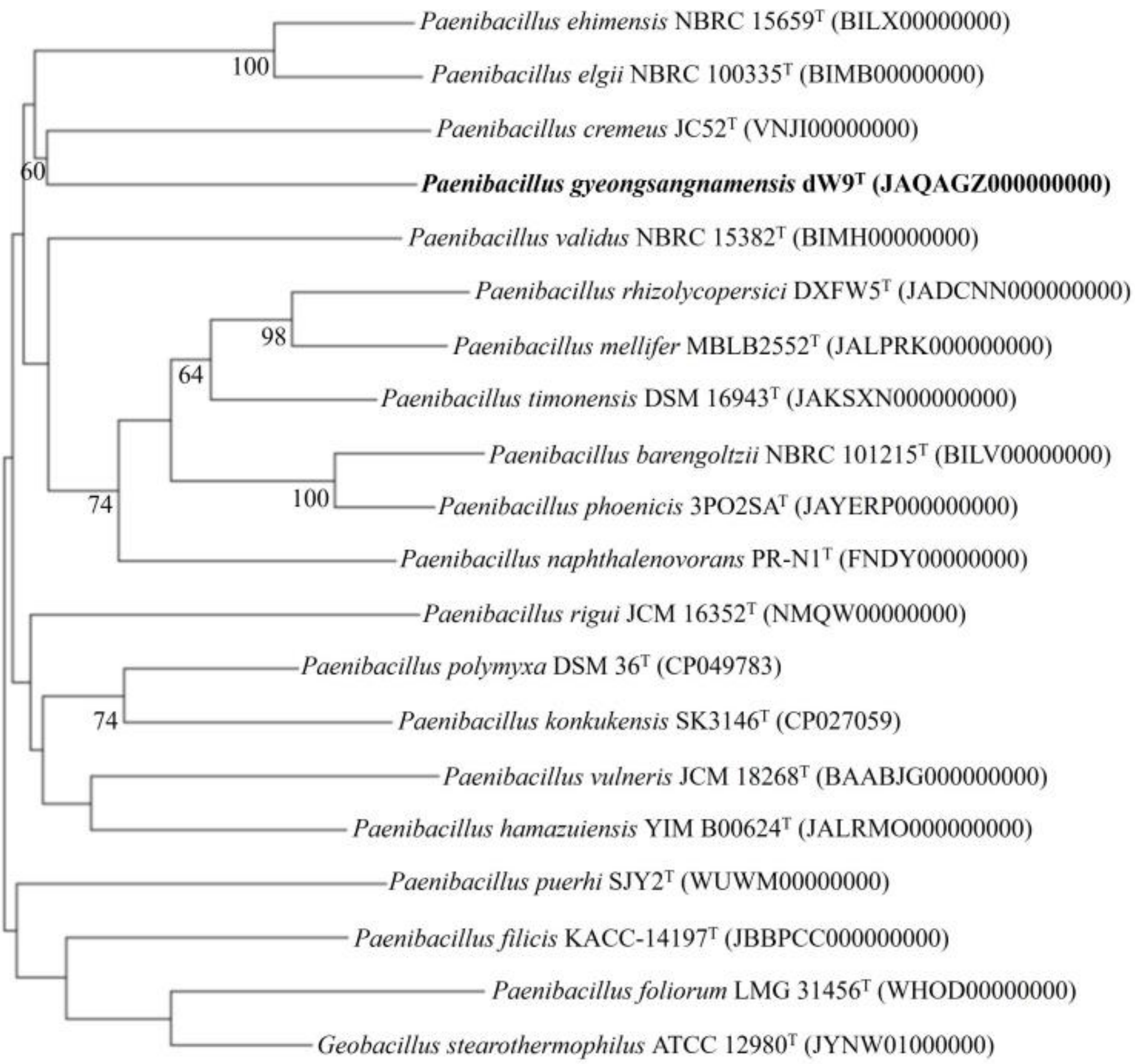

Fig. S4. Phylogenomic tree constructed with FastME 2.1.6.1 from GBDP distances calculated from genome sequence data. The numbers presented on the branches are GBDP pseudo-bootstrap support values from 100 replications.

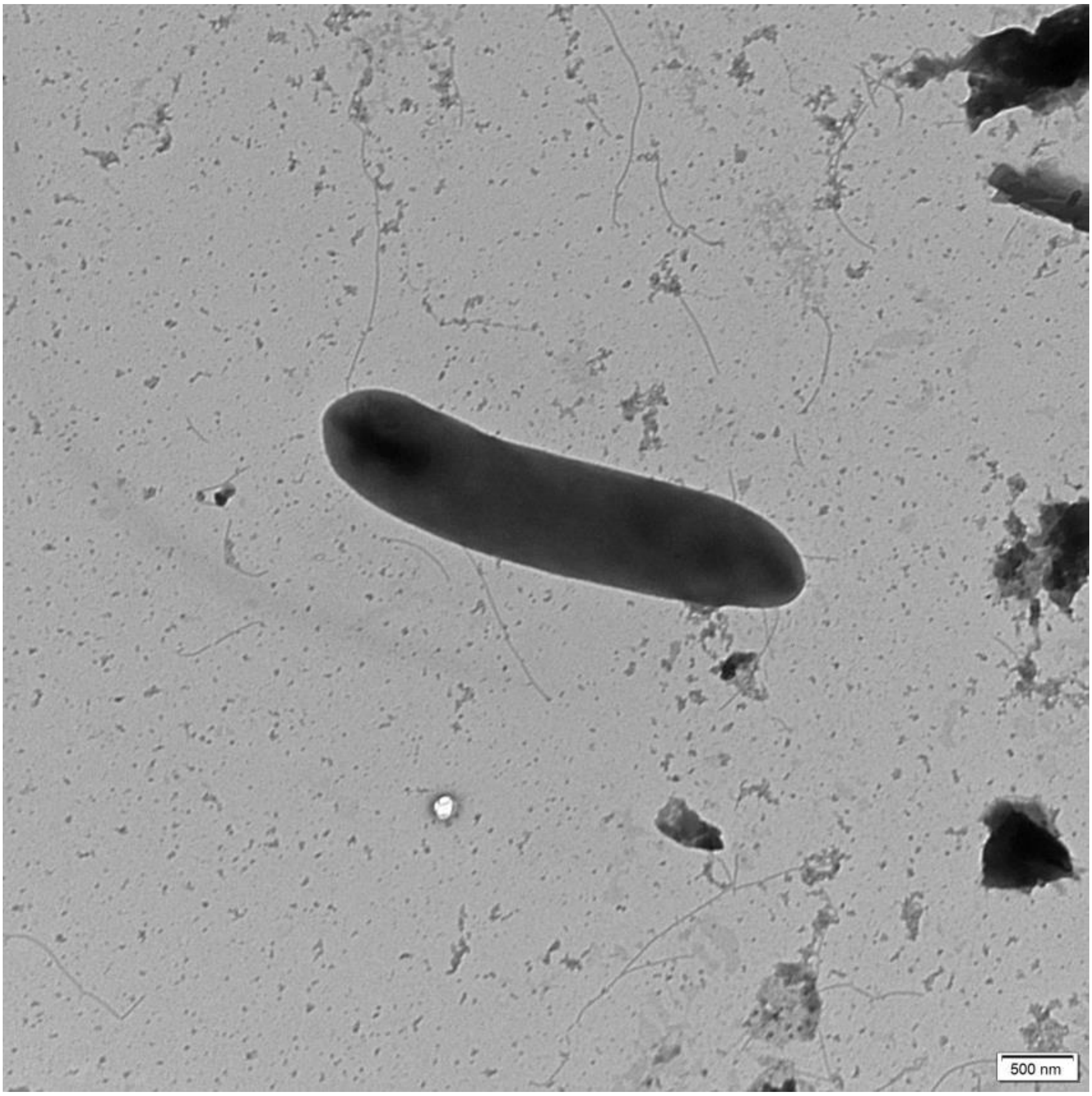

Fig. S5. Transmission electron photomicrograph of strain dW9^{T} grown on R2A agar for 5 days at 25 °C. Bars, 500 nm.

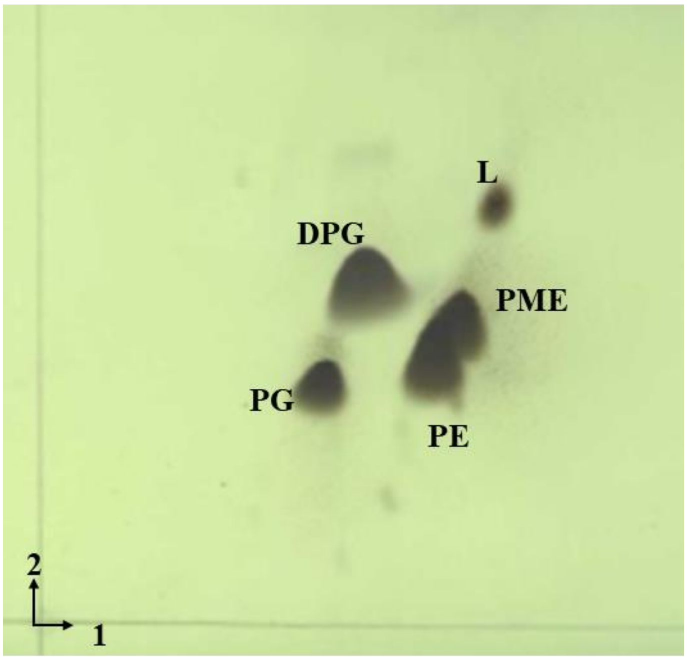

Fig. S6. Thin-layer chromatograms of the polar lipids from strain dW9^T. Abbreviations: DPG, Diphosphatidylglycerol; PG, Phosphatidylglycerol; PE, Phosphatidylethanolamine; PME, phosphatidylmethylethanolamine; and unidentified polar lipid (L).
